# Supplementary material for: The causal role of circulating inflammatory markers in osteoporosis: a bidirectional Mendelian randomized study
Source: Front Immunol. 2024 Jul 18;15:1412298. doi: 10.3389/fimmu.2024.1412298 (PMC11291241; doi:10.3389/fimmu.2024.1412298)
Supplement: Supplementary file 1 [file DataSheet_1.zip › supplementary material/CIM-OSTEOPOROSIS /GCST90274842.csv_leave_one_out.pdf]

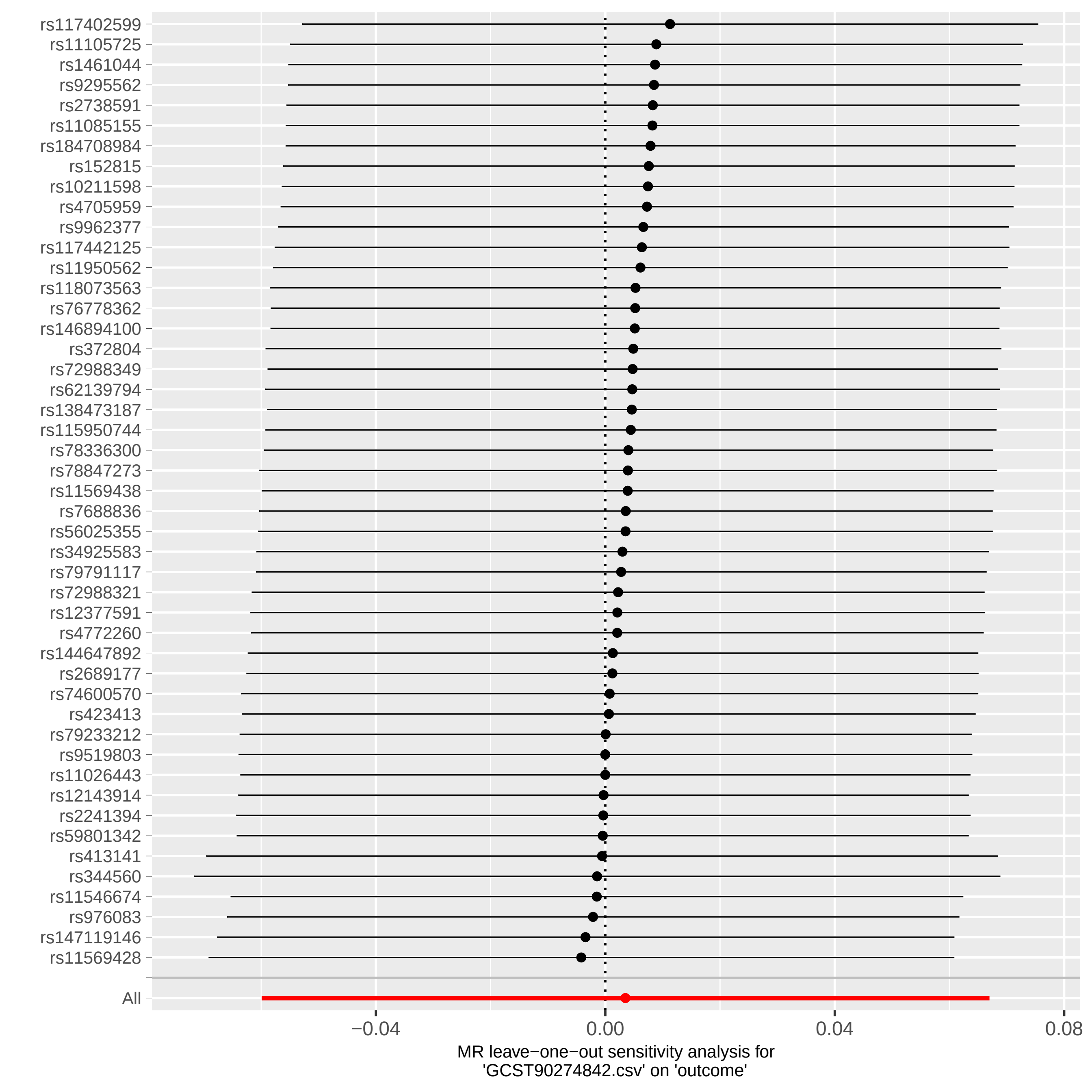
rs117402599
rs11105725
rs1461044
rs9295562
rs2738591
rs11085155
rs184708984
rs152815
rs10211598
rs4705959
rs9962377
rs117442125
rs11950562
rs118073563
rs76778362
rs146894100
rs372804
rs72988349
rs62139794
rs138473187
rs115950744
rs78336300
rs78847273
rs11569438
rs7688836
rs56025355
rs34925583
rs79791117
rs72988321
rs12377591
rs4772260
rs144647892
rs2689177
rs74600570
rs423413
rs79233212
rs9519803
rs11026443
rs12143914
rs2241394
rs59801342
rs413141
rs344560
rs11546674
rs976083
rs147119146
rs11569428
All
−0.04
0.00
0.04
0.08
MR leave−one−out sensitivity analysis for
'GCST90274842.csv' on 'outcome'
